# Effect of Surface Treated Silicon Dioxide Nanoparticles on Some Mechanical Properties of Maxillofacial Silicone Elastomer

**DOI:** 10.1155/2014/750398

**Published:** 2014-12-09

**Authors:** Sara M. Zayed, Ahmad M. Alshimy, Amal E. Fahmy

**Affiliations:** ^1^Department of Removable Prosthodontics, Faculty of Dentistry, Alexandria University, Alexandria, Egypt; ^2^Department of Dental Biomaterials, Faculty of Dentistry, Alexandria University, Alexandria, Egypt

## Abstract

Current materials used for maxillofacial prostheses are far from ideal and there is a need for novel improved materials which mimic as close as possible the natural behavior of facial soft tissues. This study aimed to evaluate the effect of adding different concentrations of surface treated silicon dioxide nanoparticles (SiO_2_) on clinically important mechanical properties of a maxillofacial silicone elastomer. 147 specimens of the silicone elastomer were prepared and divided into seven groups (*n* = 21). One control group was prepared without nanoparticles and six study groups with different concentrations of nanoparticles, from 0.5% to 3% by weight. Specimens were tested for tear strength (ASTM D624), tensile strength (ASTM D412), percent elongation, and shore A hardness. SEM was used to assess the dispersion of nano-SiO_2_ within the elastomer matrix. Data were analyzed by one-way ANOVA and Scheffe test (*α* = 0.05). Results revealed significant improvement in all mechanical properties tested, as the concentration of the nanoparticles increased. This was supported by the results of the SEM. Hence, it can be concluded that the incorporation of surface treated SiO_2_ nanoparticles at concentration of 3% enhanced the overall mechanical properties of A-2186 silicone elastomer.

## 1. Introduction

Multidisciplinary therapeutic techniques are employed in the rehabilitation of patients with advanced disease of the stomatognathic and craniofacial structures [1, 2]. Although surgical intervention can halt the disease process, prosthodontic rehabilitation is often needed to restore mastication, speech, and esthetics. Ultimately, the goals are to optimize function and cosmetic results to minimize morbidity and allow reestablishment of self-esteem [3–5].

Current materials proposed for external maxillofacial applications experience some serious problems, particularly low tear strength [6, 7]. Thus, it is necessary to have a material with satisfactory tear strength, tensile properties, and appropriate hardness. The ideal material should be similar to the missing facial tissue to optimally match a patient's articulate features of mastication, speech resonance, and facial gesture. Consequently, there is a need for improved materials with superior physical and mechanical properties that are comparable to those of human tissues and skin [8, 9]. Although numerous advances in maxillofacial prosthetic materials have been made in the past several years, the need for improvement continues [8]. Research is ongoing to develop new polymeric materials with superior mechanical properties, such as high tear strength and low hardness. A great deal of research has been devoted to developing a new class of polymeric materials by incorporating nanofillers into the organic polymer matrix, creating a nanocomposite that combines at the same time the strength of the filler and flexibility of the organic matrix [10].

Silicone elastomer has achieved a wide clinical acceptance, due to its many advantageous properties that consecrate it as the most appropriate material for facial prostheses such as biocompatibility, low chemical reactivity, ease of manipulation, and optical transparency. Furthermore, it can be pigmented to simulate skin tone; therefore, it enhances the aesthetic outcome of the prosthetic device [11, 12]. However, its mechanical properties do not fulfill the ideal requirements. The required physical and mechanical characteristics of the fabricated silicone elastomer depend on the type and the concentration of the filler used, which has to be tailored to meet the requirements of strong yet elastic material with mechanical properties that meet the clinical requirements [13–15].

Silicon dioxide nanoparticles (SiO_2_) have increasingly been exploited for numerous biomedical and biotechnological applications. Drug molecules are loaded into silica nanoparticles. Its biocompatibility makes it a benign material [16–18]. SiO_2_ nanoparticles are characterized by their small size, large interface area, active function, and strong interfacial interaction with the organic polymer [19]. Therefore, they can improve the physical, mechanical, and optical properties of the organic polymer and provide resistance to environmental stress-caused cracking and aging [20].

**Figure 1:**
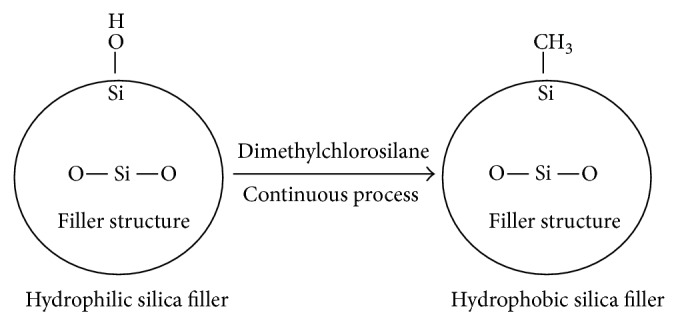
Hydrophobic silica production.

It is imperative to maintain the nanofillers content at a proper level because of their higher surface energy and chemical reactivity; otherwise, the nanoparticles may agglomerate. When the silicone elastomer is under external forces, the agglomerated particles act as stress concentrating centers in the silicone elastomer matrix, thereby decreasing the mechanical strength of the silicone elastomer [20]. Hence, it becomes crucial to incorporate well-dispersed nanofillers into the polymer to obtain beneficial mechanical and physical properties [21, 22]. Many efforts have been devoted to prevent the agglomeration and aggregation of nanoparticles, such as applying silane coupling agent between nanosilica particles and the polymer [21, 23]. Coating the inorganic filler with a silane coupling agent can link the inorganic filler and the organic matrix chemically [24]. Silica is hydrophilic due to silanol (Si-OH) groups on the surface. These silanol groups may chemically react with dimethyldichlorosilane to render the silica hydrophobic (Figure 1) [25].

Taking this as departure point, the aim of this study was to evaluate clinically the important mechanical properties of a silicone elastomer that is used for extraoral maxillofacial prosthesis after adding different concentrations of surface treated SiO_2_ nanoparticles in order to help in the design of an improved maxillofacial prosthetic material with optimum mechanical properties.

## 2. Materials and Methods

### 2.1. Specimens Preparation and Material Manipulation

147 specimens were prepared in strict compliance with the manufacturer's instructions and divided into seven groups, each of 21 specimens, such that one group (GI) was prepared without adding SiO_2_ nanoparticles to the silicone elastomer (A-2186, platinum catalyzed, vinyl terminated RTV silicone, obtained from Factor II Inc., Lakeside, AZ, USA). This will act as a control group, and six experimental groups were prepared by combining the silicone elastomer with various weight percentage amounts of the SiO_2_ nanoparticles (hydrophobic SiO_2_ coated with silane coupling agent; average particle size: 15 nm and specific surface area: 650 m^2^/g, obtained from Mknano, Mississauga, Canada), such as (GII) 0.5%, (GIII) 1.0%, (GIV) 1.5%, (GV) 2.0%, (GVI) 2.5%, and (GVII) 3.0% by weight as suggested by previous relevant study [20].

To prepare specimens for the experimental groups, SiO_2_ nanopowder was weighed by using the analytical balance (Citizen, CX 301, USA) then added to the base of the preweighed silicone elastomer gradually. The modified base was then mixed for 20 minutes using the mechanical mixer (EUROSTAR, power control-visc, IKA-Werke, Germany) at mixing speed of 150 rpm [26]. Then, the silicone cross-linking agent was added according to the manufacturer's recommended ratio of 10 : 1 by weight (base: cross-linking agent) and mixed with the modified base. The mixtures were placed in the vacuum oven (Barnstead Lab-Line, 3618-6CE, USA) for 40 minutes at pressure (948.1 mbar), as recommended by the manufacturer to get rid of the incorporated air bubbles. The vacuum was applied to a container four times the volume of the material to prevent overflow of the bubbles. The mixture was allowed to reach its maximum capacity and fall to the bottom of the container. Afterward, vacuum was held for another 5 minutes to eliminate the smaller bubbles. Eventually, the mixture was loaded into machined split copper molds lined with petroleum jelly, in specific dimensions required by each standardized test method. The material was allowed to polymerize at room temperature (23 ± 1°C) for 24 hours, after which the molds were carefully separated, specimens removed, and the flash trimmed away with a sharp scalpel. The control group was prepared in the same way as described before for the experimental groups except for adding the nanopowder.

### 2.2. Specimens Testing

All specimens were evaluated for tensile strength, percentage elongation, and tear strength using the universal testing machine (Instron 3382, USA). Shore A hardness was measured by the shore type A digital durometer (STD 226, SATRA, UK). All tests were performed at room temperature (23 ± 1°C) and relative humidity (50% ± 5%). The selection criteria of specimens for testing were absence of tears at borders, absence of air bubbles, and absence of surface irregularities.

#### 2.2.1. Tensile Strength and Percentage Elongation Testing

Seven dumbbell-shaped specimens were prepared in each group based on ASTM D412. The thickness and width of each specimen were measured at three different locations using a vernier caliper with digital readout (Absolute Digimatic Caliper, Mitutoyo, USA) and the average value was entered as input data which was used in calculating the specimen cross-sectional area via the computer software (Bluehill 2). The specimen was placed under tension in the grips of the universal testing machine and carefully adjusted symmetrically to distribute the tension equally over the cross-section. The lower member of the universal testing machine remained fixed, while the upper member moved at a constant rate of 500 mm/min cross-head speed. The maximum amount of force immediately prior to breaking (N) and elongation measurements were recorded electronically using the computer software and the resulting stress-strain curves were constructed. Tensile strength (MPa) was calculated using the following equation:

(1) $$\begin{array}{c} T_{S}=\frac{F}{A}, \end{array}$$

where *T*_*S*_ is the tensile strength (MPa); *F* is the force magnitude prior to breaking (N); *A* is the cross-sectional area of unstrained specimen (mm^2^).

The percentage elongation was calculated concurrently with the tensile strength testing. The original length was measured before testing using the digital caliper by placing benchmarks on the dumbbell-shaped specimen 25 mm apart, equidistant from the center and perpendicular to its long axis. The additional distance between the benchmarks upon sample failure, was recorded by the computer software. The percentage elongation was calculated from the equation:

(2) $$\begin{array}{c} E=100\frac{\left[L_{b}-L_{o}\right]}{L_{o}}, \end{array}$$

where: *E* is the percentage elongation; *L*_*b*_ is the length at specimen break; *L*_*o*_ is the original length.

#### 2.2.2. Tear Strength Testing

Seven trouser-shaped specimens were prepared in each group based on ASTM D624. The thickness of the specimen was measured in three different sites across the width of the specimen near its center, and the average value was recorded and entered as input data which was used in the calculations. The specimen was then placed in the grips of the universal testing machine and stretched at constant cross-head speed of 500 mm/min, until the specimen was ruptured; the force required to break the specimen (N) was recorded by the computer software. From these measurements, tear strength (N/mm) was calculated using the following equation:

(3) $$\begin{array}{c} T=\frac{F}{D}, \end{array}$$

where *T* is the tear strength (N/mm); *F* is the maximum force (N); *D* is the thickness of the specimen (mm).

#### 2.2.3. Shore A Hardness Testing

Seven specimens were prepared in each group based on ASTM D2240. Each specimen was of at least 6 mm thickness and its lateral dimensions were 12 mm from any edge. The digital durometer was placed in a vertical position and the presser foot was applied parallel to the surface of the specimens as rapidly as possible without shock. Readings were made 1 second after firm contact was achieved. Three sites were measured per each specimen, and the mean value was recorded as the hardness of each specimen.

### 2.3. Characterization of Specimens

Scanning electron microscopic (SEM) examination was performed using analytical scanning electron microscope (JSM 636OLA; JOEL, Tokyo, Japan) to monitor the dispersion of SiO_2_ nanoparticles within the silicone elastomer matrix.

#### 2.3.1. Specimens Preparation

Thin cross-sections were cut from torn tensile strength specimens and mounted rigidly on specimen holders. Since the silicone elastomer is nonconductive, accordingly the specimens were coated with an ultrathin coating of gold, by sputter coating using the ion sputtering device (JFC-1100E; JEOL, Tokyo, Japan). The gold coating is important to prevent charge build up on the specimen, but, at the same time, the thickness of the gold layer should be small enough to prevent masking of the surface layer and impairment of resolution.

#### 2.3.2. Specimens Examination

Specimens were observed at ×10,000 magnification and at an accelerating voltage of 30 KV.

### 2.4. Statistical Analysis

Data from quantitative studies of the experimental groups were collected and compared to the control group using one-way analysis of variance (ANOVA) with concentration as main variable for tensile strength, percentage elongation, tear strength, and shore A hardness. When significant differences were observed, the Scheffe test was used as post hoc test to identify differences among the groups at a significance level of *α* = 0.05 for all tests. *P* values < 0.05 were considered statistically significant. All statistical tests were performed using a statistical software SPSS (Statistical Package for Social Sciences, version 20.0, SPSS Inc., Chicago, IL, USA).

## 3. Results

**Figure 2:**
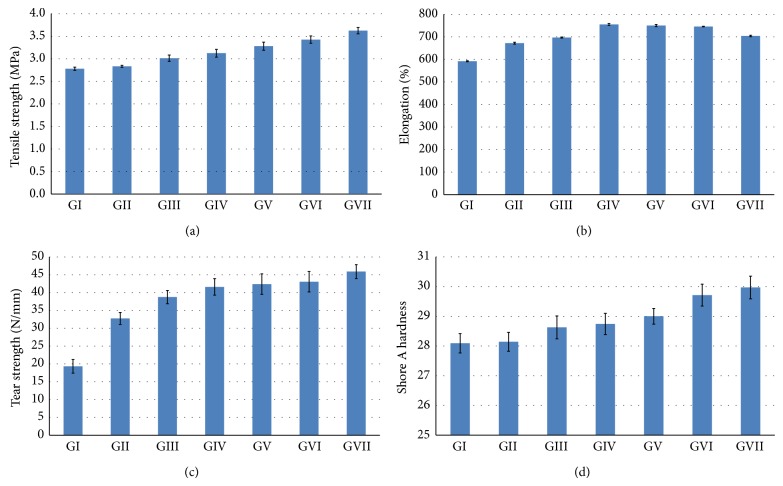
Comparative mechanical properties of the material investigated. (a) Tensile strength; (b) percentage elongation; (c) tear strength; (d) shore A hardness.

The mean values of tensile strength in (MPa) of all the studied groups containing an increasing concentration of SiO_2_ nanofiller are shown graphically in (Figure 2(a)). There was a significant increase in the tensile strength (*P* < 0.001) in the formulations prepared at 3% SiO_2_ nanofiller concentration (3.62 ± 0.69 MPa) when compared with that of the control group (2.78 ± 0.36 MPa). There was no significant difference (*P* > 0.05) in the tensile strength of formulations prepared at 0% and 0.5% concentration.

The mean values of percentage elongation of all the studied groups containing an increasing concentration of SiO_2_ nanofiller are shown graphically in (Figure 2(b)). The greatest value was in the 1.5% formulation (754.8 ± 4.06), and there was a small but significant decrease in the percentage elongation as the concentration of SiO_2_ nanofiller increased from 2 to 3%.

The mean values of tear strength in (N/mm) of all the studied groups containing an increasing concentration of SiO_2_ nanofiller are shown graphically in (Figure 2(c)). The tear strength of the formulations prepared at 3% SiO_2_ nanofiller concentration (45.90 ± 1.94 N/mm) was significantly (*P* < 0.001) greater than that of the control group 0% (19.32 ± 1.90 N/mm).

The mean values of shore A hardness of all the studied groups containing an increasing concentration of SiO_2_ nanofiller are shown graphically in (Figure 2(d)). There was no significant difference (*P* > 0.05) in the hardness of formulations prepared at 0% and 0.5%. There was also no significant difference when the SiO_2_ nanofiller increased from 0.5 to 1.5%, and there was no statistically significant difference between the 2.5% and the 3% SiO_2_ nanofiller concentration. There was a small but significant increase in the hardness as the concentration of the SiO_2_ nanofiller was increased from 0% (28.09 ± 0.32) to 3% (29.97 ± 0.38).

**Figure 3:**
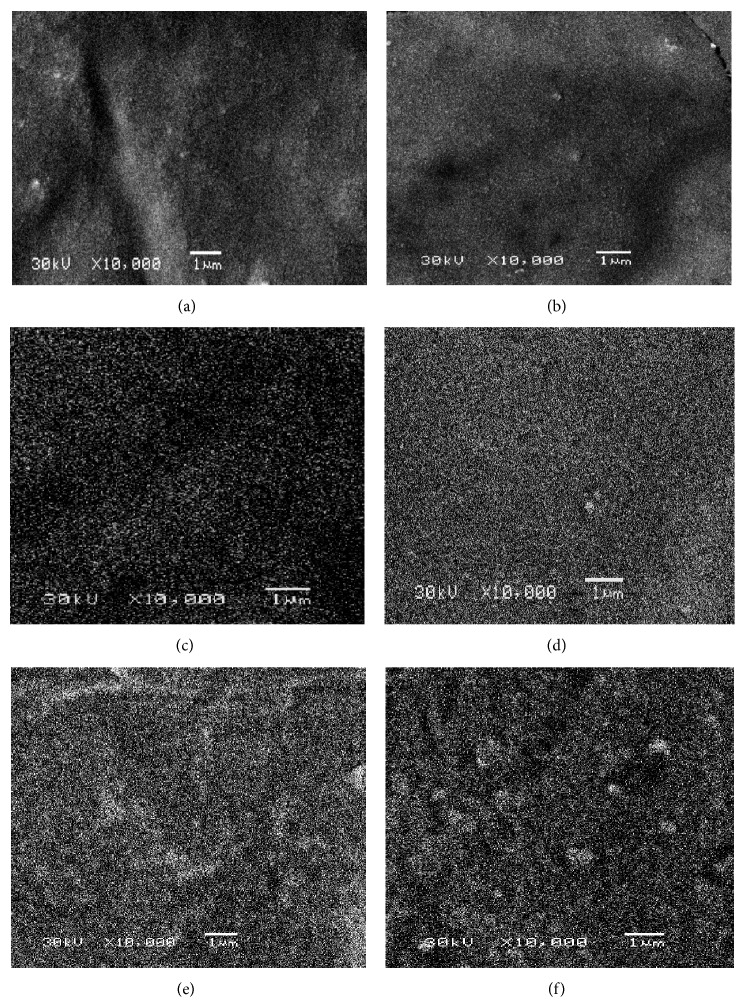
SEM images showing the homogenous dispersion of the nano-SiO_2_ (×10,000). (a) 0.5% nano-SiO_2_ (GII); (b) 1% nano-SiO_2_ (GIII); (c) 1.5% nano-SiO_2_ (GIV); (d) 2% nano-SiO_2_ (GV); (e) 2.5% nano-SiO_2_ (GVI); (f) 3% nano-SiO_2_ (GVII).

SEM images demonstrate the homogenous dispersion of the spherical and whitish SiO_2_ nanoparticles within the silicone elastomer specimens as shown in (Figure 3). SEM examination indicated that all the nano-SiO_2_ concentrations were distributed uniformly throughout the silicone specimens. No aggregates were detected as the SiO_2_ nanoparticles loading was increased in all specimens.

## 4. Discussion

The aim of this study was to develop an improved maxillofacial prosthetic material with optimum mechanical properties. The main focus was to enhance the tensile strength, tear strength, and the percentage elongation. To accomplish this, formulations were developed by incorporating different concentrations of surface treated SiO_2_ nanofiller, followed by evaluation of the mechanical properties, in view of the fact that testing of the mechanical properties is an important step towards the modification of the current material or acceptance of a new material.

Incorporation of silica (SiO_2_) filler into a silicone polymer is called compounding [27]. This is accomplished prior to cross-linking. The addition of silica filler is a vital factor in the physical and mechanical properties of silicone elastomers, because the unfilled cross-linked polydimethylsiloxane (PDMS) has very low mechanical properties, since a very high cross-link density produces an inelastic brittle material [11, 27]. That made using silica fillers essential for enhancing the mechanical properties. The addition of surface treated silica fillers can augment the tensile strength of the cross-linked polymer by up to 40 times [25]. The reason behind the increased strength is the strong physical and chemical bonds between the vulcanized polymer and the silica filler. Thus, polymer/filler interactions are maximized [26, 28, 29]. The hydrophobic surface treatment of the filler is also essential to prevent water absorption into the cured PDMS elastomer, since finished facial prostheses is subjected to sebum, sebaceous, and perspirations from the underlying living human skin which may lead to deterioration of the prosthesis [30].

Surface treated silica fillers are also better at dispersion into the silicone elastomer and have a reduced base viscosity compared to nonsurface treated silica fillers. Under deformation, these surface treated fillers help to increase the strength of the elastomer by allowing the polymer chains to uncoil and slide past neighboring filler particles increasing the crystallization between neighboring PDMS chains [26]. However, clinically acceptable mechanical properties are only achieved at the correct filler concentration.

As depicted in the statistical analysis, results of this study revealed significant improvement in the tensile strength and tear strength with the use of 3% concentration of surface treated SiO_2_ nanofiller, given that the values of the experimental group (GVII) were found to be significantly higher than those of the control group (GI). The percentage elongation was found to increase with increasing the nanofiller concentration till reaching its maximum value at 1.5% (GIV) then started to decrease with increasing the concentration of the nanofiller but still the 3% has a higher value of elongation than the control group. This should not pose any serious problem, because clinically the value of the elongation obtained is accepted as satisfactory for the use of maxillofacial prosthesis. The improvement in the mechanical properties may be attributed to the enhanced polymer adsorption by the nanosilica's large surface area. Moreover, the higher surface energy and chemical reactivity of the nanoparticles allowed them to interact with the silicone elastomer matrix and form a 3-dimensional network by chemical bonding in the presence of the surface treatment [31, 32]. By this means, silicone elastomer with high tear strength, tensile strength, and elongation percentage is produced. A high percentage elongation and high tear strength produce the most desirable combination [27, 33]. The surface treatment furthermore improved the incorporation and dispersion of the nano-SiO_2_ in the silicone elastomer matrix; this was supported by the SEM images which revealed uniform dispersion of the nano-SiO_2_ in specimens of all the six concentrations tested.

Results revealed as well a small but significant increase in the hardness as the concentration of the nanofiller increased from 0.5 to 3% but still within the clinically acceptable range (25–35 shore A). As described in the literature [34, 35], higher filler loading may result in further increase in the hardness.

The study performed by Han et al. [20] recommended the incorporation of nanooxides of Ti, Zn, or Ce (nonsurface treated) at concentrations of 2 to 2.5% by weight into A-2186 silicone elastomer; these concentrations improved the mechanical properties. Their results indicated that when the concentration was increased to 3%, the tear strength, tensile strength, and elongation decreased. Contrasting with this, the results of the present study indicated that with the use of 3% surface treated nano-SiO_2_, there was more improvement in the tear strength, tensile strength, and elongation. SEM performed by Han et al. [20] revealed that, at a concentration of 2%, particles of all the three nanooxides were well distributed in the silicone elastomer matrix. However, when the concentration was increased to 3%, all the three nanooxides had agglomerated which resulted in a decrease in the mechanical properties of silicone elastomer. On the other hand, results from the present study demonstrated that, with the use of 3% surface treated nano-SiO_2_, SEM showed no particles agglomeration and thus improvement in the mechanical properties; this may be attributed to the surface treatment of the nanoparticles.

According to the results of the present study, the surface treated nano-SiO_2_ filler evenly dispersed within the silicone matrix and consequently improved the mechanical properties of the silicone elastomer. Therefore, it is possible to affirm that, apart from the increase in filler/polymer interaction, the surface treatment could improve the dispersion of nanosilica within the silicone matrix by reduction in silica agglomeration, which was supported by the SEM images. The interaction between the original filler and the surface treated silica is still questionable. It is proposed that they interact with each other by van der Waals force and hydrogen bonds [25].

The addition of the surface treated SiO_2_ nanofiller in 3% concentration did not affect the viscosity of silicone base. A further increase in filler concentration may lead to filler overloading, producing the highest base viscosity. In other words, the increase in loading of silica might result in a difficulty in the mixing process. The addition of that nanofiller did not also influence the translucency of silicone elastomer considerably, contrasting with the fact that the addition of the same concentration (3%) of nano-TiO_2_ turned the specimen white. Accordingly, the present study recommends the use of 3% surface treated nano-SiO_2_, since the concentration is appropriate for reinforcing the mechanical properties of maxillofacial silicone elastomer (A-2186) without much affecting the hardness, the translucency, or the viscosity.

Future work should be planned to investigate the effect of surface treated nano-SiO_2_ on the ability to provide skin-colored prostheses and its color stability. The effect of artificial accelerated weathering and the influence of chemical disinfection on the mechanical and physical properties of silicone elastomer modified with surface treated nano-SiO_2_ require evaluation as well.

## 5. Conclusions

Under the conditions of this study and with the specific materials used, the following conclusions can be derived.

1. The incorporation of surface treated SiO_2_ nanoparticles for the reinforcement of maxillofacial silicone elastomer A-2186 provided it with more favorable mechanical properties, especially in terms of tear strength.
2. Surface treatment of the SiO_2_ nanoparticles improved its distribution within the silicone matrix and prevented its agglomeration.

## Clinical Significance

The increase in tensile strength, tear strength, and percentage elongation of the maxillofacial silicone material provides a clinical advantage to the marginal integrity of a facial prosthesis. Thus, the esthetic quality of a facial prosthesis will be improved by permitting a thinner margin with a greater possibility of stretching and less possibility of tearing.
